# Intersectional research on dementia care for post-migrants and ethnic minority groups: a scoping review

**DOI:** 10.3389/frdem.2025.1596395

**Published:** 2025-08-18

**Authors:** Kübra Altinok, Seda Güney, Bugse Yüceer, Oliver Razum, Martina Roes

**Affiliations:** ^1^German Center for Neurodegenerative Diseases (DZNE), Witten, Germany; ^2^School of Public Health, Bielefeld University, Bielefeld, Germany; ^3^Faculty of Nursing Science, Koç University, Istanbul, Türkiye; ^4^Gulhane Health Science Institute, University of Health Sciences, Ankara, Türkiye; ^5^Department of Nursing Science, Faculty of Health, University of Witten/Herdecke, Witten, Germany

**Keywords:** dementia, intersectionality, preferences, ethnic minority group, post-migrants, diversity

## Abstract

**Objective:**

The aim of this scoping review is to identify the range, extent and nature of evidence available in peer-reviewed and gray literature and to examine how the intersecting experiences and differences of post-migrants and ethnic minority groups influence preferences.

**Methods:**

The Arksey and O’Malley methodological framework and the PRISMA-ScR for Scoping Reviews confirm the rigor of the scoping review. We systematically searched across electronic databases including PubMed, MEDLINE via Ovid, CINAHL, Scopus, Cochrane Library as well as gray literature between December 2023 and September 2024. We included the articles in English, German, and Turkish languages without any publication date restrictions. We analyzed the identified intersectional determinants of preferences using inductive content analysis.

**Results:**

Limited studies focusing on the intersectional determinants of preferences of ethnic minority groups or post-migrants with dementia were found. After screening 1,404 studies, we identified 4 relevant papers through the search strategy. Additionally, we identified 18 records through hand-searching, gray literature, and reference list checks. In total, 22 articles were included in this review. Inductive content analysis allowed to reach six main-theme focusing on the preferences, which are preferences connected to informal care providers, expectations in nursing homes/care institutions, preferences of care setting, coping approaches and preferences, food preferences, and other daily preferences.

**Discussion:**

Our findings highlight the complexity of personal care preferences, showing that expectations and practices are shaped by cultural values, traditions, language barriers, immigration processes, shifting socioeconomic positions, and gender roles. The lack of attention to the preferences of the diverse groups and the limited support for their unique wishes and needs indicates a significant challenge in the health system. Caregiving choices and daily living are shaped by intersecting structural determinants and differences. However, there are still gaps in research, healthcare practices, and public awareness, making it harder to meet their preferences.

**Systematic review registration:**

Registration DOI: https://doi.org/10.17605/OSF.IO/Z8T9H.

## 1 Introduction

As of 2020, it was estimated that more than 50 million individuals worldwide were living with dementia, and this number is expected to triple by 2050 (Nichols et al., 2022). Over 500.000 individuals with a migrant history in European countries have been diagnosed with dementia (Monsees et al., 2021). There are significant disparities between people with and without a migration history because of differentials in socioeconomic background, health literacy, culture, psychological well-being, and language proficiency (Kovaleva et al., 2021).

Despite these substantial numbers, the lived experiences of post-migrants and ethnic minority groups with dementia remain insufficiently explored (Low et al., 2019; Gove et al., 2021; Motta-Ochoa et al., 2021). In addition, culturally sensitive medical and nursing care as well as multilingual information materials have not yet been standardized (BMFSFJ, 2020). In some cases, organizational ideas about culture-sensitive regulations and person-centered care (PCC) do not match the social reality of people with dementia (du Toit and Buchanan, 2018). This gap highlights the critical need for culturally and linguistically sensitive, accessible, and inclusive care services. It also underscores the importance of integrating diverse perspectives into global strategies that address dementia to understand and address the nuanced impacts of structural and social factors on dementia care (Chejor et al., 2022; Roes et al., 2022). Failure to adopt such inclusive approaches may perpetuate the invisibility of certain social groups within nursing and dementia research. Individuals from diverse groups face challenges such as, language barriers, knowledge gaps, and sociocultural and economic hardships (Taiebine et al., 2023; Siette et al., 2023; Chejor et al., 2023). These challenges can hinder effective communication with healthcare professionals, complicate accurate diagnosis and treatment and increase the risk of discrimination by nursing staff (Chejor et al., 2023; Migala and Flick, 2020). Stigma and marginalization can further restrict access to healthcare services, because some cultural groups may view dementia as a sign of weakness, which may discourage individuals from seeking help (Siette et al., 2023; Zhao et al., 2023). Cultural norms and traditional health beliefs may also lead to the perception of dementia as a natural part of aging, making early diagnosis difficult. Post-migrants and ethnic minority groups may experience multiple forms of discrimination due to their intersecting social identities, such as gender, age, education level, and migration status, which influence their wishes and needs (Liu et al., 2008; Hossain and Khan, 2020; Wong et al., 2024). These factors often result in unmet needs and reduced quality of care for these diverse groups.

To effectively manage dementia and enhance the well-being of individuals with dementia from diverse groups, it is crucial to understand their beliefs, expectations, and preferences with regard to the dementia process. PCC is essential because, it ensures that individual preferences and needs are recognized and addressed appropriately, which may lead to more effective and equitable care (Kitwood and Bredin, 1992). This approach requires an understanding of preferences, personalities, and habits and the integration of these factors into daily care (Fazio et al., 2018; Van Haitsma et al., 2020; Fazio et al., 2018; Van Haitsma et al., 2020). This may improve access to healthcare services, encourage active participation in the care process, and enhance the satisfaction and quality of life for post-migrants and ethnic minority groups with dementia (Fazio et al., 2018; Cabote et al., 2023).

According to Van Haitsma et al. (2020) preferences encompass various behaviors and activities of daily life, such as leisure activities, care and support, social interaction, and routine daily activities. Consideration of the preferences of diverse groups in dementia care allows for the delivery of more sensitive and effective care (Van Haitsma et al., 2020). For example, ensuring that a person’s dietary preferences align with his or her cultural beliefs or that social activities are presented in the person’s native language and cultural context helps in tailoring care to these preferences (Chejor et al., 2023; Cabote et al., 2023; Rosendahl et al., 2016). Similarly, language is not only a tool of communication but a signifier of individuality, community and cultural belonging reflecting sociocultural values such as hierarchy or gender roles (Junghare, 2013).

Understanding the preferences, needs, and values of post-migrants and ethnic minority groups with dementia requires recognition of their complex identities and unique and varied characteristics (Kassam et al., 2020; Sagbakken et al., 2018a). To address these complexities, the perspective of intersectionality is useful for understanding the overlapping identity components of individuals and the interaction of these components in shaping personal experiences and needs. Intersectionality discourse was introduced by Crenshaw in 1989 to analyze inequities within axes of racism and sexism to understand Black and Indigenous women’s experiences (Crenshaw, 2013). The discourse underlines the interconnectedness and interplay of constructs such as race, class, and gender as interdependent systems of power (Kassam et al., 2020). For example, defining gender requires moving beyond the binary view of sex, gender, and sexuality, as these identities are performative and always intersect with other factors (Butler, 1990; Czapka and Sagbakken, 2020). From a structural intersectionality perspective, social identities and categories are formed by social interaction, power dynamics, and system of oppression (e.g., structural racism, structural sexism, and classism) (Homan et al., 2021).

In connection with the intersectionality perspective, it is important to deconstruct the categorization of migrants/minorities while utilizing alternative terms that do not contribute to the re/production of inequalities. The categorization of migration status mostly involves dichotomous classifications such as involuntary/voluntary, legal/illegal, and minority/majority people with/without a migration background (Robertson, 2019). These terms, such as “migrants/immigrants,” “migration background” and “second generation,” create a hierarchy and false sense of homogeneity and intensify the demarcation between “us” and “them” (Borrelli and Ruedin, 2024). Researchers also tend to use generalized terms for culture or ethnicity despite the aim of amplifying the voices of these diverse groups. Similarly, the terms “culture” and “ethnicity” frame migrants and their descendants as “ethnic others” (Römhild, 2017). This culturalized concept of difference leads to “integration” approaches that position migrants as “minorities” who must assimilate or tolerate them (Römhild, 2017). However, the solution is not to avoid the use of these terms but rather to introduce a well-considered terminology that proposes a flexible, context-specific approach and to be self-reflective about the terms while using them (Borrelli and Ruedin, 2024; Römhild, 2017). In this study, we adopt the terms “ethnic minority groups” and “post-migrants.” The term “minority group” appears to refer to a population subgroup that is numerically smaller than the majority within a given culture, but this definition does not include being politically disempowered (Blume, 2013). A minority group member is someone who identifies with or is labeled as part of a minority that, due to differing worldviews, often faces oppression from the majority (Blume, 2013). One of our focuses is minority groups defined by ethnicity, although it is important to acknowledge the existence of other minority groups on the basis of gender, sexual orientation, or disability. Ethnicity can be understood as a dynamic and multidimensional social construct shaped by interactions between individuals, groups, and broader societal structures (Barth, 1969). Rather than viewing ethnic groups as carriers of fixed cultural traits, perceiving ethnicity as fluid is important to realize its construction through boundary-making process within a specific social context (Barth, 1969).

Another focus is “post-migrants” with the aim of capturing the complex sociopolitical dynamics that form migration experiences and shape the collective transformation of society through the migration process (Yurdakul, 2024). The prefix “post” here does not indicate the end of migration but underlines social negotiation processes that occur during the phase after migration has occurred (Foroutan, 2015). The post-migrant perspective attempts to deconstruct the “either/or” logic that perceives migration as a deviation from the imagined settled white citizen (Barbarino and Seydel, 2024). It acknowledges the lasting impact of immigration and emigration and recognizes that these changes form the social structure in a country.

People living with dementia from post-migrants and ethnic minority groups may have different care preferences shaped by many overlapping experiences. These include what happened before migration, such as trauma, limited access to education, or low health literacy (Stypińska and Gordo, 2018). The migration process itself can bring challenges like insecure legal status, language difficulties, or losing connection to community and culture (Stypińska and Gordo, 2018). After moving to a new country, they may experience racism, barriers accessing healthcare, or navigating different cultural and generational identities (Ahmad et al., 2022). Studies of the experiences of people with a migration history who have dementia have focused mostly on the need for culture-specific offers (Cabote et al., 2023). However, the complex experiences of the group are overlooked in the adaptation of this one-sided determinant. Since culture is not homogeneous, it is important to embrace a perspective considering multiple identities of individual intersecting with the structural inequalities in the health system (Homan et al., 2021; Lewis et al., 2021; Monsees et al., 2020).

The development of approaches to dementia care that account for this diversity is crucial for improving the quality of life of individuals and fostering a more inclusive healthcare system (WHO, 2021). The development of PCC strategies necessitates a comprehensive understanding of the preferences of this population, which are influenced by their multidimensional identities (Roes et al., 2022; Kassam et al., 2020). To increase the integration of this underrepresented group into the healthcare system and to provide PCC, it is essential to recognize their preferences and understand the intersectional factors that influence these preferences (Roes et al., 2022; Van Haitsma et al., 2012). With this goal, we conducted a scoping review to categorize the extent of available evidence within peer-reviewed and gray literature. Our research questions were as follows: “What are the multilayered experiences of post-migrants and ethnic minority groups with dementia and their caregivers?” and “How are their preferences in dementia care influenced by their intersecting experiences?”

## 2 Materials and methods

### 2.1 Study design

We used the PRISMA guidelines (Tricco et al., 2018) and the step-by-step framework for scoping studies established by Arksey and O’Malley and refined by Levac et al. (2010). This framework suggests six main stages: (Nichols et al., 2022) defining the research question, (Monsees et al., 2021) finding relevant studies, (Kovaleva et al., 2021) selecting studies, (Low et al., 2019) organizing the collected data, (Gove et al., 2021) summarizing and presenting the findings, and (Motta-Ochoa et al., 2021) seeking input from others. In reporting our inclusion and exclusion criteria for the scoping review (Table 1), we considered the PCC (population, concept of interest, and context) metric (Pollock et al., 2023). The scoping methodology was selected for this review to provide a comprehensive and systematic overview of the intersectional determinants of the preferences of post-migrants and ethnic minority groups living with dementia. This approach enables the inclusion of diverse study designs to foster a broader understanding of research in this area. The protocol was developed prior to the beginning of the review and was registered by OSF registries on July 17, 2024 (Altinok et al., 2025). The submitted protocol was followed except that we have not conducted the consultation step with people with dementia, their caregivers and healthcare providers to the due to time constraints and limited stakeholder resources.

**Table 1:** Inclusion and exclusion criteria.

Criteria	Definition
Population	We included studies that involved post-migrants with dementia and individuals from minority groups with varied care needs across different healthcare settings without specific country restrictions. Additionally, we included studies in which caregivers, professionals, or family members provided proxy reports of the preferences of the group living with dementia. Studies that focused on individuals who were receiving palliative care or studies that focused on end-of-life preferences were excluded from this review.
Concept of Interest	We reviewed studies that detailed the preferences of diverse groups who experienced dementia from an intersectional perspective. To ensure comprehensive coverage of relevant literature, we expanded our search to include related terms and synonyms for “preferences” even when these terms were not explicitly stated in the titles or abstracts. During the full-text screening process, we considered multiple terms for the inclusion or exclusion criteria rather than limiting our focus solely to “preferences.” The following terms were used in this context: Preferences Expectations Wishes Needs Values Demands Goals Attitudes
Context	The intersectional determinants of preferences were the primary focus of this review. This included a broad range of factors, such as personality traits, life experiences, overall health, and sociocultural background in addition to structural determinants such as ethnicity, gender, socioeconomic status, education, citizenship, and geographical location.
Types of evidence sources	The search strategy focused on peer-reviewed empirical research articles. All types of studies were considered for inclusion. Systematic reviews, discussion papers, mission statements, conference abstracts, editorials, and articles without full-text reports were excluded. Additionally, gray literature (dissertation & thesis) was included. We excluded preprints.
Other	We included studies published in English, German and Turkish. There were no restrictions on publication date.

### 2.2 Search strategy and study selection

We searched for peer-reviewed journal articles and gray literature published in English, German and Turkish and uploaded the articles to the web-based software platform Covidence (2024). We conducted searches across electronic databases, including PubMed, MEDLINE (via Ovid for precision and controlled searching), CINAHL (via EBSCO), Scopus, and the Cochrane Library, between December 2023 and September 2024. Moreover, we conducted backward and forward citation tracking from June–September 2024 by examining reference lists and utilizing Google Scholar and ResearchGate. The search terms were developed in alignment with our research questions (Table 2). The inclusion and exclusion criteria were adjusted at the beginning of the screening process. Two reviewers (KA and SG) screened the titles and abstracts of all records and reported the screening process using the PRISMA flowchart (Tricco et al., 2018). The full texts were also independently reviewed for eligibility by the same two reviewers, and the reasons for exclusion were carefully documented. Any discrepancies between the reviewers were addressed through discussion. If consensus could not be reached, the coauthors (OR and MR) were consulted to resolve the issue.

**Table 2:** Search strategy.

Key words	Databases	Records
*Population*: Alzheimer* OR Dementia* OR Elderly*OR Older* OR Senior* OR “Dementia” OR cognitive impairment OR cognitive dysfunction OR cognitive decline AND immigrant* OR origin* OR migrant* OR migration OR background OR ethnic* OR ethnoc* OR ingroups OR outgroups OR kinship OR “minority group*” OR “minority population*” OR minorities OR multicultu* OR polyethnic* OR latin* OR polyethnic* OR “afro american*” OR black OR roma OR romani OR “migrant” OR “people of color” OR native	Cochrane Library	942
	PubMed	307
	CINAHL	75
	MEDLINE via Ovid	12
	Web of Science	131
*Context*: preference OR expectation OR wish OR need OR value OR demand OR goal OR attitude	Scopus	41
*Concept*: intersection* OR intersecting OR multilayered OR gender OR caste OR sex* OR race OR ethnicity OR norm OR class OR religion OR disability OR weight OR physical appearance OR education OR colour OR color OR aboriginality OR migration status OR visa status OR language OR religion OR ability OR age OR mental health OR socioeconomic OR housing status OR geographic location OR medical record OR criminal record OR cultur* OR biological OR biology OR body OR BAME OR BAMER OR sociocultural OR health status OR health issues OR stigma OR discrimination OR history OR location OR place OR divers* Or difference OR inequalit* OR community OR underrepresented OR disparit* OR multicultur* OR intercultur*	Gray Literature Hand Search (Google Scholar & Research Gate), and Reference Lists	18

### 2.3 Charting the data

We extracted details about the studies’ characteristics, such as the study design, language, and publication type. Population characteristics (i.e., sample size and information about post-migrants and ethnic minority groups with dementia) and setting information (i.e., healthcare context, country of origin, and study period) were documented in addition to the aims of the studies.

### 2.4 Collating, summarizing and reporting the results

The articles included in this study were analyzed via qualitative content analysis with an inductive approach (Vears and Gillam, 2022) using MAXQDA software (VERBI Software, 2022). This analysis involved *in vivo* coding (verbatim representation) and descriptive coding (summarizing the meaning of extracted text into words or concise phrases) to comprehensively assess the included studies (Rivas, 2012). We also summarized the analyses through the lens of intersectionality and the characteristics of preferences. First, to provide an intersectional perspective, we included articles that considered the complex identities of individuals and experiences of differences (Hancock, 2016). We focused on the preferences changing according to multidimensionality and co-occurrence of multiple positions (Liu et al., 2022). The complexity of considering co-occurrence of multiple identities and axes of inequality together is always a challenge, which requires the inclusion of macro- and micro-level analysis simultaneously (Homan et al., 2021). However, we tried to capture as many individual and structural categories as possible while focusing on their interplay with each other. The data analysis process was initially conducted by a primary researcher (KA) and subsequently cross verified by secondary researchers (SG and MR). Upon completion of data extraction, the same two reviewers (KA and SG) re-examined each extracted item to ensure consistency. If consensus was not achieved, the coauthors (OR/MR) were consulted for resolution.

## 3 Results

### 3.1 Search results

**Figure 1:**
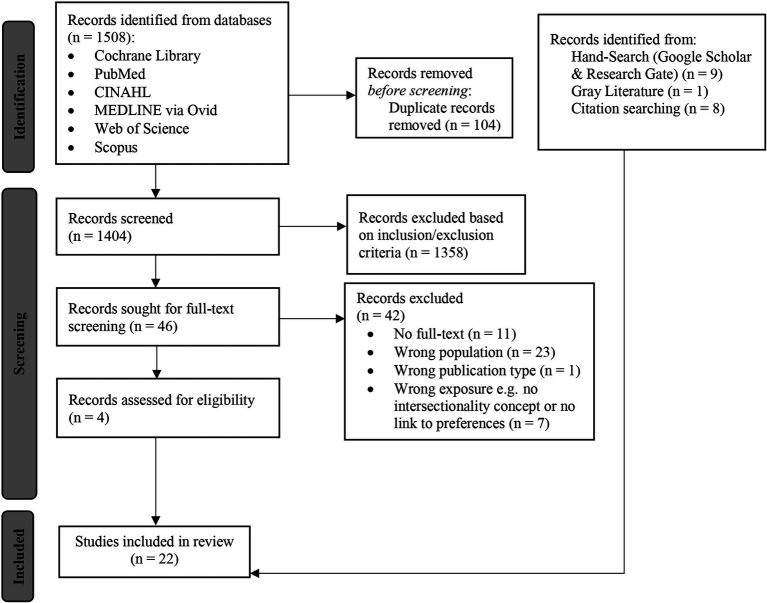
PRISMA flow chart.

The database searches were initially conducted in September 2023 across five databases. The initial results generated a total of 1,404 articles after the removal of duplicates, of which 4 were included in the review. Through citation tracking and hand searches, we identified another 18 articles. In total, we included 22 articles in this review. Among the 22 included articles, 21 were peer-reviewed journal articles, while one was a doctoral dissertation (Figure 1).

### 3.2 Study characteristics

The studies (see Table 3) were from the following countries: the US (Liu et al., 2008; Lewis et al., 2021; Dilworth-Anderson and Gibson, 2002; Im et al., 2022; Martinez et al., 2022; Nkimbeng et al., 2022; Richardson et al., 2019; Kong et al., 2010; Lee Casado et al., 2015), Australia (Ramsay et al., 2017; Shanley et al., 2012), Belgium (Chaouni et al., 2024), Canada (Wong et al., 2024), Germany (in German) (Tezcan-Güntekin, 2018), Norway (Sagbakken et al., 2018a; Czapka and Sagbakken, 2020; Sagbakken et al., 2020; NÆSs and Moen, 2014), Poland (Leszko and Allen, 2024), Sweden (Antelius and Plejert, 2016), and the UK (Hossain and Khan, 2020; Lawrence et al., 2011). Most were in English, with the exception of one dissertation in German. The studies were published between 2002 and 2024. Most of the studies were qualitative and used semi-structured/in-depth interviews and focus group interviews. There was one cross-sectional survey (Im et al., 2022) and one mixed-method study (Nkimbeng et al., 2022). The status of migration history or the information about the minority group is expressed in the same way that it was mentioned in the articles (e.g., immigrants, migrants, Alaskan Indians, Latinos). The studies involved participants from different groups: African Americans in the US (Dilworth-Anderson and Gibson, 2002; Im et al., 2022; Nkimbeng et al., 2022; Richardson et al., 2019), Alaskan Indian people in Alaska (Lewis et al., 2021), Chinese communities in the US (Liu et al., 2008; Dilworth-Anderson and Gibson, 2002) Australia (Shanley et al., 2012), and Canada (Wong et al., 2024); Europeans in the US (Dilworth-Anderson and Gibson, 2002); Hispanic groups in the US (Dilworth-Anderson and Gibson, 2002; Im et al., 2022; Richardson et al., 2019); Asian groups in the US (Im et al., 2022), and the UK (Lawrence et al., 2011); Latino communities in the US (Martinez et al., 2022), Polish groups in the US (Leszko and Allen, 2024), and Norway (Sagbakken et al., 2018a; Sagbakken et al., 2020); Korean Americans in the US (Richardson et al., 2019; Kong et al., 2010; Lee Casado et al., 2015), South Asians in Canada (Wong et al., 2024), the Spanish-speaking communities in Australia (Shanley et al., 2012) and Canada (Wong et al., 2024); Italian-speaking groups in Australia (Shanley et al., 2012) and Belgium (Chaouni et al., 2024), Arabic-speaking groups in Australia (Shanley et al., 2012), Middle Eastern group in Sweden (Antelius and Plejert, 2016); Turkish community in Belgium (Chaouni et al., 2024), Germany (Tezcan-Güntekin, 2018) and Norway (Ramsay et al., 2017); Bangladeshi group in the UK (Hossain and Khan, 2020), Vietnamese community in the US (Liu et al., 2008) and Norway (Sagbakken et al., 2020); Pakistani group in Norway (Sagbakken et al., 2018a; Czapka and Sagbakken, 2020; Sagbakken et al., 2020; NÆSs and Moen, 2014); and people from various countries including India, Afghanistan, Iran, Algeria, Mexico, Chile, Lebanon, Sri Lanka and Bosnia in Norway (Sagbakken et al., 2018a; Sagbakken et al., 2020).

**Table 3:** Study characteristics.

Author Details, Year, Institution, and Country	Method applied	Publication type and language	Sample and sample size	Population and country they live in ^a^
1. Antelius and Plejert (2016) Dementia caregiving targeted towards Middle Eastern immigrants living in Sweden. Linköping University, Sweden	In-depth interviews	English, peer-reviewed article	*N* = 10 care staff	Middle Eastern immigrants^b^ living in Sweden
2. Chaouni et al. (2024) The influence of religion on the care experiences of family carers of older migrants with dementia in Belgian cities. Vrije Universiteit, Belgium	In-depth interviews	English, peer-reviewed article	*N* = 34 family caregivers	Migrant group with Moroccan, Turkish, and Italian descent with dementia living in Belgium
3. Czapka and Sagbakken (2020) “It is always me against the Norwegian system.” barriers and facilitators in accessing and using dementia care by minority ethnic groups in Norway: a qualitative study. Oslo Metropolitan University, Norway	Semi-structured in-depth interviews	English, peer-reviewed article	*N* = 8 families *N* = 5 key representatives of immigrant communities *N* = 6 representatives of health and care personnel	Participants from Somalia, Poland, Croatia, Pakistan, India, Turkey and one of the islands in the Atlantic Ocean living in Norway
4. Dilworth-Anderson and Gibson (2002) The Cultural Influence of Values, Norms, Meanings, and Perceptions in Understanding Dementia in Ethnic Minorities. The University of North Carolina at Greensboro, USA	Secondary data analysis from structured and semi-structured interviews	English, peer-reviewed article	*N* = 32 caregivers	Caregivers, self-identified as being of African American, Chinese American, European American, and Hispanic (Puerto Rican, Dominican, or Hispanic) descent living in the US
5. Hossain and Khan (2020) Barriers to access and ways to improve dementia services for a minority ethnic group in England. Keele University, the UK	Phase (1) Focus group interviews Phase (2) Semi-structured interviews	English, peer- reviewed article	Phase (1) *N* = 21 general members of the Bangladeshi community Phase (2) *N* = 6 Bangladeshi family caregivers	Bangladeshi people living in U.K.
6. Im et al. (2022) Attitudes toward Alzheimer’s disease and dementia caregiving and health outcomes: Racial and ethnic differences. Nell Hodgson Woodruff School of Nursing, Emory University, USA	Cross-sectional online survey	English, peer-reviewed article	*N* = 172 family caregivers of persons living with AD	African Americans (*n* = 41), Hispanics (*n* = 40), and Asian groups (*n* = 55) living in the US
7. Kong et al. (2010) The Experience of Family Caregivers of Older Korean Americans With Dementia Symptoms. University of Maryland, USA	Semi-structured focus group interview	English, peer-reviewed article	*N* = 23 family caregivers	Korean Americans living in the US
8. Lawrence et al. (2011) Threat to Valued Elements of Life: The Experience of Dementia Across Three Ethnic Groups. King’s College London, the UK	In-depth interviews	English, peer-reviewed article	*N* = 30 people with dementia	Black Caribbean and South Asian people with dementia living in U.K.
9. Lee Casado et al. (2015) The Experience of Family Caregivers of Older Korean Americans With Dementia Symptoms. University of Maryland, USA	Focus group interviews	English, peer-reviewed article	*N* = 23 family caregivers	Korean Americans living in the US
10. Leszko and Allen (2024) Caring From a Distance: Experiences of Polish Immigrants in the United States Providing Care to Parents With Dementia Overseas. University of Szczecin, Szczecin, Poland	Semi-structured interviews	English, peer-reviewed article	N = 37 caregivers	Polish Immigrants living in the US
11. Lewis et al. (2021) “Making Sense of a Disease That Makes No Sense”: Understanding Alzheimer’s Disease and Related Disorders Among Caregivers and Providers Within Alaska Native Communities. University of Minnesota Medical School, USA	Semi-structured interview	English, peer-reviewed article	*N* = 21 caregivers *N* = 15 health care providers	Alaska Natives (ANs) caregivers and ANs and non-native health care providers living in Alaska
12. Liu et al. (2008) Re-examining the relationships among dementia, stigma, and aging in immigrant Chinese and Vietnamese family caregivers. UC Davis Medical School, USA	In-depth interviews	English, peer-reviewed article	*N* = 32 family caregivers	Chinese and Vietnamese group living in the
13. Martinez et al. (2022) The Experience of Alzheimer’s Disease Family Caregivers in a Latino Community: Expectations and Incongruences in Support Services. California State University Long Beach, USA	In-depth interviews	English, peer-reviewed article	*N* = 24 caregivers *N* = 10 service providers	Latino Community living in the US
14. NÆSs and Moen (2014) Dementia and migration: Pakistani immigrants in the Norwegian welfare state. Oslo and Akershus University College of Applied Sciences, Norway	Field observations and in-depth interviews	English, peer-reviewed article	*N* = 22 families and healthcare employees	Norwegian-Pakistanis living in Norway
15. Nkimbeng et al. (2022) The Immigrant Memory Collaborative: A Community–University Partnership to Assess African Immigrant Families’ Experiences with Dementia. University of Minnesota School of Public Health, USA	Phase (1) Community-Based Participatory Research; Phase (2) semi-structured focus group interview; Phase (3) survey	English, peer-reviewed article	Phase (1) *N* = 20 community stakeholders for the participatory research Phase (2) *N* = 24 African immigrants for community conversation Phase (3) *N* = 170	African Immigrant living in the US
16. Ramsay et al. (2017) Migrant caregiving for family members with mild cognitive impairment: an ethnographic study. The University of Melbourne, Australia	In-depth interviews with ethnographic approach	English, peer-reviewed article	*N* = 10 caregivers	Turkish caregivers living in Australia
17. Richardson et al. (2019) At the intersection of culture: Ethnically diverse dementia caregivers’ service use. Ohio State University, USA	In-depth interviews	English, peer-reviewed article	*N* = 15 caregivers	Hispanic, African American, and South Korean participants living in the US
18. Sagbakken et al. (2018a) Dementia and Migration: Family Care Patterns Merging With Public Care Services. The Norwegian Centre for Migration and Minority Health (NAKMI), Norway	In-depth interviews and dyad interviews	English, peer-reviewed article	*N* = 12 relatives *N* = 18 health personnel	Immigrants from Pakistan, India, Afghanistan, Iran, Turkey, Algeria, Mexico, Chile, Poland, and Bosnia living in Norway
19. Sagbakken et al. (2020) How to adapt caring services to migration driven diversity? A qualitative study exploring challenges and possible adjustments in the care of people living with dementia. Oslo Metropolitan University, Norway	Individual and dyad interviews, and focus groups discussions	English, peer-reviewed article	*N* = 19 single interviews, *N* = 3 dyad interviews, and *N* = 16 focus groups with older immigrants, relatives of immigrants with dementia, and health personnel	Older immigrants and their relatives/family members from culturally and linguistically various background (Pakistan, India, Afghanistan, Iran, Turkey, Algeria, Mexico, Chile, Poland, and Bosnia, China, Vietnam, Lebanon, and Sri Lanka) living in Norway
20. Shanley et al. (2012) A qualitative study into the use of formal services for dementia by carers from culturally and linguistically diverse (CALD) communities. University of New South Wales, Australia	Focus group interviews, one-to-one interviews	English, peer-reviewed article	*N* = 121 family carers, *N* = 60 health professionals	Italian, Chinese, Spanish and Arabic-speaking communities living in southwestern Sydney, Australia
21. Tezcan-Güntekin (2018) Stärkung der Selbstmanagement-Kompetenzen pflegender Angehöriger türkeistämmiger Menschen mit Demenz^c^. Bielefeld University, Germany	Expert interviews	German, dissertation	*N* = 11 experts in the medical/social work/nursing fields, N = 12 family caregivers	Turkey-origin people living in Germany
22. Wong et al. (2024) Living with dementia: Exploring the intersections of culture, race, and dementia, stigma. The University of British Columbia, Canada	Semi-structured interviews	English, peer-reviewed article	*N* = 10 people living with dementia	South Asian, Spanish-speaking and Chinese living in the Vancouver, Canada

a The articles included study populations comprising either people with dementia, caregivers of people with dementia, or care staff working with individuals with dementia. In addition, some studies involved key representatives of immigrant communities, healthcare and care personnel, and community stakeholders.

b We used the terminology used for post-migrant and ethnic minority groups as presented in the reviewed articles.

c [Strengthening self-management skills of family caregivers of Turkey-origin people with dementia].

### 3.3 Description of the preferences of the diverse group members

Our results provide an interpretative analysis of the intersectional determinants of the groups’ preferences. Six main themes were discussed from an intersectional perspective (see Table 4): *preferences of care setting, expectations in nursing homes/care institutions, preferences connected to informal care providers, coping approaches and preferences, food preferences, and other daily preferences.*

**Table 4:** Intersectional determinants and preferences in care contexts.

Preferences	Intersecting determinants	Key insights	References
Care setting preferences	Age Culture Gender Class Migration	The discussion involves preferences between home care or nursing home as well as the role of intersectional categories and differences playing the role on this decision. Post-migrants and minority groups tend to choose home care over nursing homes.	Hossain and Khan (2020), Czapka and Sagbakken (2020), Martinez et al. (2022), Nkimbeng et al. (2022), Richardson et al. (2019), Kong et al. (2010), Ramsay et al. (2017), Chaouni et al. (2024), Tezcan-Güntekin (2018), Sagbakken et al. (2020), NÆSs and Moen (2014), Leszko and Allen (2024), and Antelius and Plejert (2016)
Expectations in nursing homes/care institutions	Gender Generation Religion Migration Language	The main focus of the theme is on wishes, needs, expectations in the nursing homes, adult daycare services, and other care facilities. Several expectations include language-sensitive and culture-sensitive services, as well as respect for traditional habits.	Hossain and Khan (2020), Czapka and Sagbakken (2020), Richardson et al. (2019), Kong et al. (2010), Shanley et al. (2012), Tezcan-Güntekin (2018), Sagbakken et al. (2020)
Preferences connected to informal care providers	Class Ethnicity Gender Age	The preferences expected by the informal care providers are influenced by intersectional determinants. In most diverse communities, it is common for younger family members to support older adults with dementia.	Dilworth-Anderson and Gibson (2002), Im et al. (2022), Martinez et al. (2022), Lee Casado et al. (2015), Tezcan-Güntekin (2018), Lawrence et al. (2011)
Food preferences	Culture Religion, Tradition Migration	The food preferences are shaped by several structural differences. The studies discuss the necessity for nursing home to consider these needs.	Wong et al. (2024), Czapka and Sagbakken (2020), Richardson et al. (2019), Shanley et al. (2012), Sagbakken et al. (2020)
Coping approaches and preferences	Culture Religion, Tradition Migration	Alternative preferences such as plant-based medicines, essential oils, music were discussed to cope with dementia.	Hossain and Khan (2020), Lewis et al. (2021), Richardson et al. (2019), Chaouni et al. (2024)
Daily preferences	Generation Spirituality Class Gender Age	Everyday-living preferences involve needs and expectations related to sleeping habit, bathing arrangements, dressing, housing, and life styles. The intersecting categories shape these preferences in various ways.	Hossain and Khan (2020), Wong et al. (2024), Sagbakken et al. (2018a), Lewis et al. (2021), Richardson et al. (2019), Kong et al. (2010), Chaouni et al. (2024), Sagbakken et al. (2020)

The extracted data are presented in Supplementary Table 1, where the general characteristics of the determinants, such as type, topics and preferences, are summarized. These topics can be categorized as follows.

#### 3.3.1 Preferences intersecting with the care setting

Fourteen studies included in the review discussed preferences for the care setting in relation to various structural determinants, such as culture, gender, migration, generation, age, education, class, religion and ethnicity (Hossain and Khan, 2020; Czapka and Sagbakken, 2020; Martinez et al., 2022; Nkimbeng et al., 2022; Richardson et al., 2019; Kong et al., 2010; Lee Casado et al., 2015; Ramsay et al., 2017; Shanley et al., 2012; Chaouni et al., 2024; Tezcan-Güntekin, 2018; Sagbakken et al., 2020; Leszko and Allen, 2024; Antelius and Plejert, 2016). The discussions focused mostly on decisions between homecare and nursing homes for post-migrants and minority group members with dementia in various communities. The preference for homecare was evident for Turkish, Hispanic, African American, Bangladeshi, Pakistani, Korean, Middle Eastern, and Polish groups and diverse communities in Norway (Hossain and Khan, 2020; Martinez et al., 2022; Nkimbeng et al., 2022; Richardson et al., 2019; Lee Casado et al., 2015; Ramsay et al., 2017; Shanley et al., 2012; Tezcan-Güntekin, 2018; Leszko and Allen, 2024; Antelius and Plejert, 2016), whereas the desire for care provision in nursing homes was considered in diverse communities in Norway and Belgium as well as Hispanic and Polish communities in the US (Czapka and Sagbakken, 2020; Martinez et al., 2022; Richardson et al., 2019; Chaouni et al., 2024; Sagbakken et al., 2020; Leszko and Allen, 2024). The included studies revealed the complexity of intersecting determinants, which shaped the needs, preferences, expectations and attitudes of post-migrants and ethnic minority groups with dementia and their family caregivers.

The intersection of culture, migration and generational differences played a significant role in the preference for a care setting. Caregiving was perceived as a family responsibility and cultural necessity between generations. Family values remain an important factor influencing the choice of care setting. Especially when considering broader definitions of family that go beyond blood relations and includes close friends, social networks and communities (often defined ‘chosen family’) (Dilworth-Anderson and Gibson, 2002). The studies strongly emphasized the understanding of filial piety as family members often actively chose family-based care to maintain traditional values in the group (Richardson et al., 2019; Kong et al., 2010; Lee Casado et al., 2015; Tezcan-Güntekin, 2018). For example, Ramsay et al. revealed that for Turkish caregivers living in Western countries, traditional values related to filial responsibilities are deeply influential and often prevented families from considering formal healthcare options (Ramsay et al., 2017). In this cultural context, formal caregiving options were not perceived as a support system; rather, they were associated with moral failure, abandonment and disrespectfulness (Hossain and Khan, 2020; Nkimbeng et al., 2022). Therefore, families tended to choose homecare, which often resulted in the younger generation taking responsibility for older adults.

For many caregivers and family members, migration strengthened their preferences for the homecare setting. However, these preferences showed generational differences and gender aspects. Women often took the primary caregiving responsibility as a way of honoring familial and cultural obligations in a migratory context (Shanley et al., 2012). However, when it comes to second-generation, caregivers may adopt both cultural expectations and the norms of the society in which they live (Czapka and Sagbakken, 2020; Tezcan-Güntekin, 2018). For example, Czapka and Sagbakken (2020) indicated that second-generation immigrants did not perceive caring for older adults as solely women’s responsibility. Similarly, Tezcan-Güntekin (Tezcan-Güntekin, 2018) discussed the generational shift and role of women, which is also shaped by the urban and rural context and parents’ wishes for their children to maintain their care.

The decision-making process regarding the care setting are shaped significantly by the intersection of culture, social class, and education (Martinez et al., 2022; Kong et al., 2010; Shanley et al., 2012; Tezcan-Güntekin, 2018; Leszko and Allen, 2024). Leszko and Allen (2024) emphasized that for many Polish immigrants with lower financial resources in the US, the choice of home care is not solely a cultural preference but also a necessity considering the high cost of nursing homes. Families often cannot afford the additional costs of day care facilities or nursing homes, making these options impractical (Martinez et al., 2022; Tezcan-Güntekin, 2018; Leszko and Allen, 2024). However, there are contradictory examples from other cultures. In China, community services are connected to stigma because people use these services only if they do not have sufficient family support or are struggling financially (Kong et al., 2010). Therefore, even if they are economically stable, they tend to reject nursing homes because of the stigma and prejudgment. Most Chinese-speaking people in Australia are therefore reluctant to receive health services (Shanley et al., 2012). In Hispanic groups living in the USA, low education levels and unfamiliarity with the healthcare system have shaped the preference for homecare, which made them to be hesitant about using care institutional care services, such as day care services (Martinez et al., 2022).

Another important factor that intersects with culture is religion. The decision about the care setting has a significant connection with culturally and religiously aligned services (Hossain and Khan, 2020; Richardson et al., 2019; Ramsay et al., 2017; Shanley et al., 2012; Chaouni et al., 2024; Sagbakken et al., 2020; NÆSs and Moen, 2014). Chaouni et al. (2024) reported that for many older migrants with dementia, the ability to continue religious customs, such as praying, wearing a headscarf, consuming halal food, or cleaning habits, greatly impacts their acceptance of professional care services. Hossain and Khan (2020) discussed concerns about a lack of sensitivity to key religious traditions, which results in anxiety about unmet needs in institutional care. According to the perspective of family members, providing care to older adults at home is connected to Islamic teachings and beliefs, cultural practices, customs, and the values of Bangladeshi Muslim family caregivers (Hossain and Khan, 2020). For Turkish families living in Australia, it is perceived as a sin to place elders into residential care (Ramsay et al., 2017). In the Norwegian–Pakistani community, younger generations feel a strong moral obligation to provide familial care to older relatives with dementia (NÆSs and Moen, 2014). These studies exemplify the deep-rooted cultural value of intergenerational reciprocity intensified by the religious understanding of caring for older adults. Religion, which intersects with language and culture, is also considered a core aspect of the preference for care institutions. The studies also reported that people with dementia and their family caregivers perceived shared language not only as a form of communication but also in relation to religious and cultural beliefs (Hossain and Khan, 2020; Sagbakken et al., 2020).

Different perspectives on the care system also lead to different expectations depending on structural intersecting factors. These viewpoints often involve expectations of care institutions and professional healthcare providers.

Nine articles analyzed preferences for care institutions from an intersectional perspective (Hossain and Khan, 2020; Martinez et al., 2022; Richardson et al., 2019; Kong et al., 2010; Lee Casado et al., 2015; Shanley et al., 2012; Chaouni et al., 2024; Tezcan-Güntekin, 2018; Sagbakken et al., 2020). Language seems to be a foundational aspect of effective dementia care for post-migrants and ethnic minority groups with dementia (Richardson et al., 2019; Kong et al., 2010; Tezcan-Güntekin, 2018). The studies indicated that individuals with dementia and their family care providers preferred healthcare professionals who spoke the same language, which fostered cultural understanding (Richardson et al., 2019; Kong et al., 2010). Shanley et al. (2012) indicated that bilingual workers from ethnospecific or multicultural care facilities provided more personalized and culturally sensitive care because of their linguistic and cultural parallelism with people with dementia.

Gender critically shapes preferences regarding cultural sensitivity in care institutions. Kong et al. (2010) described a case in which a Korean woman resisted undressing for a bath in front of health personnel, which resulted in aggressive behavior. However, the study indicated that the wishes of residents could be fulfilled by respecting traditional habits. The study highlighted the importance of culturally and gender-sensitive care in nursing homes and care institutions to provide comfort to residents (Kong et al., 2010). Shanley et al. (2012) similarly shows that Arabic community members, especially Muslims, saw religious beliefs as a barrier to using services that involved being cared for by someone of the opposite gender or sharing spaces like group settings or sleeping areas with the opposite gender. Some studies have identified a hierarchy of priorities with regard to preferences for care settings that balance religion, gender, culture and ethnicity. Bangladeshi family caregivers, for example, expressed the highest priority for same-sex Muslim caregivers. When this ideal match was not available, they prioritized gender over religious or ethnic dimensions and accepted non-Muslim but same-sex healthcare providers for their relatives with dementia in care institutions (Hossain and Khan, 2020).

#### 3.3.2 Preferences intersecting with the dyad of the person with dementia and family caregiver

Fifteen of the included studies provided an understanding of the interconnectedness of cultural values, age factors and generational differences (Liu et al., 2008; Sagbakken et al., 2018a; Dilworth-Anderson and Gibson, 2002; Im et al., 2022; Martinez et al., 2022; Richardson et al., 2019; Kong et al., 2010; Lee Casado et al., 2015; Ramsay et al., 2017; Shanley et al., 2012; Tezcan-Güntekin, 2018; NÆSs and Moen, 2014; Leszko and Allen, 2024; Antelius and Plejert, 2016; Lawrence et al., 2011). The concept of filial piety, including the need to provide care to family members, was common in many studies, including Asian communities; Hispanic groups; African–American communities; Chinese, Turkish, Arabic, and Polish communities; Korean communities; Italian and Spanish speaking communities; and Pakistani communities (Dilworth-Anderson and Gibson, 2002; Im et al., 2022; Martinez et al., 2022; Richardson et al., 2019; Kong et al., 2010; Shanley et al., 2012; Tezcan-Güntekin, 2018; Sagbakken et al., 2020; NÆSs and Moen, 2014; Leszko and Allen, 2024). The studies discussed the need for family care and the responsibility of younger family members to provide care to older adults.

This complexity is intensified by the intersecting factors of culture and gender. The studies discussed the intergenerational gender roles that determined the preferences of people with dementia (Richardson et al., 2019; Kong et al., 2010; Lee Casado et al., 2015). In most cases, wives, daughters, or daughters-in-law were seen as the main caregivers (Hossain and Khan, 2020; Im et al., 2022; Martinez et al., 2022; Richardson et al., 2019; Kong et al., 2010; Lee Casado et al., 2015; Ramsay et al., 2017; Shanley et al., 2012; Tezcan-Güntekin, 2018). However, migration history, acculturation and generation alter life conditions and gender roles. The studies mentioned that the second generation often continued to care for their parents and maintained traditional practices, whereas the third generation, which was influenced by the process of migration and acculturation, tended to change these customs (Dilworth-Anderson and Gibson, 2002; Richardson et al., 2019). The studies explained this trend through the evolving dynamics of cultural identity and the increasing participation of women in the workforce (Dilworth-Anderson and Gibson, 2002; Richardson et al., 2019; Kong et al., 2010).

In addition, religion was mentioned as a determinant of preferences for informal care providers (Chaouni et al., 2024). Caring for an older relative with dementia was a natural reflex shaped by religious and cultural values, as cited in Islamic texts and traditions (Chaouni et al., 2024).

Some post-migrants and ethnic minority groups with dementia and their informal caregivers showed a preference for traditional healing practices and spiritual approaches (Hossain and Khan, 2020; Lewis et al., 2021; Richardson et al., 2019; NÆSs and Moen, 2014). Across these studies, caregivers described the use of methods such as plant-based medicines, essential oils, music, and social engagement to create a holistic caregiving experience and to embrace cultural traditions and values. The studies indicated the significance of spirituality and religion as coping mechanisms. For example, participants from Bangladeshi Muslim communities emphasized the role of Islamic teaching and values in guiding their caregiving approach (Hossain and Khan, 2020). Religious practices such as prayer, mass, and religious hymns were helpful as sources of comfort and relief (Chaouni et al., 2024). These activities were considered beneficial both when they were practiced individually and when they were shared with older people with dementia. The study also emphasized that visiting a mosque or church provided comfort and a sense of identity for religious older people with a history of migration (Chaouni et al., 2024). Spiritual practices and holistic perspectives, such as praying, meditation, and communal religious activities, were considered methods to enhance individuals’ social, spiritual, emotional, communal, and cultural well-being (Hossain and Khan, 2020; Lewis et al., 2021). For example, in the Hispanic community, maintaining traditional practices such as nightly prayers and the use of candles provided comfort (Richardson et al., 2019). Additionally, the studies indicated a preference for combining traditional healing practices with Western medical treatments (Lewis et al., 2021; NÆSs and Moen, 2014). While Western medicine was considered helpful for the management of specific symptoms, the integration of traditional healing methods involved culturally meaningful practices and beliefs for diverse group members (NÆSs and Moen, 2014).

#### 3.3.3 Preferences intersecting with everyday life

The studies revealed that the daily preferences of people with dementia are complex and interconnected and should be considered in relation to other elements to provide personalized care. Five articles included in the review identified intersectional determinants in relation to the daily preferences of post-migrants and ethnic minority groups with dementia (Wong et al., 2024; Richardson et al., 2019; Kong et al., 2010; Chaouni et al., 2024; Tezcan-Güntekin, 2018). Kong et al. (2010) reported that for Korean immigrants with dementia, daily preferences were connected to culture and tradition, such as sleeping arrangements, food preferences and lifestyles. Generational differences were also relevant in shaping these preferences; the way people eat, dress, and live is influenced by acculturation or traditional Confucian values, even if they have lived in a new country for years (Kong et al., 2010). Older generations who had lived in the US for years tended to maintain traditional practices. They may feel more attached to traditional Korean meals and modes of sleeping, such as sleeping on the floor rather than in a bed.

One of the main topics discussed in the studies was the observation of intersectional determinants of food preferences. For post-migrants and ethnic minority groups with dementia, food often implies traditional and cultural familiarity, sometimes with religious obligations, cultural history, daily routines, or identity shaped by migration history (Hossain and Khan, 2020; Wong et al., 2024; Lewis et al., 2021; Richardson et al., 2019; Kong et al., 2010; Lee Casado et al., 2015; Shanley et al., 2012; Chaouni et al., 2024; Tezcan-Güntekin, 2018; Sagbakken et al., 2020). As individuals with dementia grow older, traditional foods play a vital role in preserving their cultural identity (Wong et al., 2024; Kong et al., 2010).

The studies emphasized not only the importance of traditional food but also the necessity of approaching people with dementia by considering their complex needs in relation to food. For example, in focus group discussions, many participants in nursing homes highlighted the importance of culturally and religiously appropriate food, such as their wish for halal meals (Shanley et al., 2012).

Regarding personal activities such as bathing, it is possible to observe the influence of cultural and gender-specific values (Kong et al., 2010). For example, a Korean female resident with dementia in a nursing home felt uncomfortable when she had to disrobe in front of others before bathing, which clashed with traditional Korean values (Kong et al., 2010).

The interconnectedness of cultural components and religion also plays a significant role in preferences (Hossain and Khan, 2020; Richardson et al., 2019; Chaouni et al., 2024). For example, hygiene practices among religious Bangladeshi Muslims remain important and must be followed according to religious rules (Hossain and Khan, 2020). Chaouni et al. (2024) mentioned a Turkish person with dementia insisted on dressing outside a bathroom because it clashed with their hygiene values and habits, suggesting that the perception of hygiene is shaped by religious and cultural factors.

## 4 Discussion

This review explored the complex intersecting factors that influence the preferences, wishes, needs, attitudes and expectations of post-migrants and ethnic minority groups with dementia. Few studies adopted an intersectional perspective; therefore, our findings provide a more interpretative approach to understanding these multilayered experiences.

We found that the preferences, wishes, expectations or needs of post-migrants and minority group members with dementia is interconnected and fluid. Post-migrants and ethnic minority groups may have similar wishes and expectations. However, when we consider other intersecting categories and determinants in the subgroups, the preferences become more complex. For example, when it comes to caring a person with dementia, some studies examine experiences through single dimension, such as gender, and conclude that women are affected the most in these case; however, intersectional approach recognizes that factors like sociocultural context, financial conditions, migration, or ethnicity interact to shape experiences of people with dementia and caregivers (Liu et al., 2022). By considering these overlapping influences, the outcomes shift and the expected result of burden in female caregivers, for example, decreases in black families in comparison to white families (Liu et al., 2022; Dilworth-Anderson et al., 2020). In addition, the experiences in subgroups seems similar, which demonstrates the recognition of differences helps us to understand vulnerability in the groups and the unique types of preferences with different sets of gender, migration, class, age, ethnicity, generation, tradition, religion, culture etc. (Sagbakken et al., 2020).

The preferences, expectations, attitudes, needs or wishes of people living with dementia were mentioned only in very few studies (Wong et al., 2024; Czapka and Sagbakken, 2020; Richardson et al., 2019; Tezcan-Güntekin, 2018). Most studies focused on the decisions regarding homecare or nursing homes. However, only a few studies provided information about preferences in one of these care settings. Most studies examined food, hygiene, sleeping preferences and religious and spiritual practices. However, several needs were not discussed. For example, sexuality was discussed in only one article (Tezcan-Güntekin, 2018). This does not mean that this group has no sexual needs; rather, it presents a critical observation of why such needs are neither considered nor addressed within the group, particularly when cultural taboos and gender are taken into consideration, which is important because it emphasizes the lack of focus on this area in research (Horne et al., 2021).

The role of traditional foods in maintaining the cultural identity and familiarity of post-migrants and ethnic minority groups with dementia reflects the intersection of cultural norms, religious values and migration history (Kong et al., 2010). Studies have shown that food preferences evolve with age and the progression of dementia and that these preferences are a crucial element of care. The results also highlight broader cultural influences on daily preferences, such as sleeping habits, personal hygiene and lifestyle choices. These preferences may be influenced by cultural values even after years of acculturation (Kong et al., 2010). Spirituality and religion emerged as significant coping mechanisms among individuals from CALDwD and offered both emotional support and a framework for caregiving. The preference for integrating traditional healing practices with Western medical treatments similarly mediated the integration of cultural and spiritual perspectives (Lewis et al., 2021).

Several of the reviewed articles did not directly discuss the preferences of individuals with dementia; however, they provided a broader perspective that directly or indirectly influenced the preferences of post-migrants and ethnic minority groups with dementia (Jaldin et al., 2023). Terms such as filial piety or familism were very common in the articles. Filial piety is a cultural value rooted in various societies, such as Korean, Latinx and Chinese communities (Monsees et al., 2020; Lee Casado et al., 2015; Jaldin et al., 2023). It implies a cultural expectation that adult children will take care of their older parents that is passed down from generation to generation (Lee Casado et al., 2015). In some groups, for example, African-Americans or Chinese-Americans, the concept of family extends beyond the household to the wider community, making the care a collective responsibility (Dilworth-Anderson and Gibson, 2002; Bullock et al., 2003). Caregiving often involves not only close relatives but also extended family and non-kin helpers (Bullock et al., 2003). This sometimes put many pressure on informal caregivers because they do not only prove daily care and support their loved ones with dementia but they also play an active role in formal care systems that do not always meet their needs. In many cases, family members have to step in as translators and cultural brokers because formal caregivers do not speak the same language or understand important cultural practices (Plejert et al., 2017). In the example of Middle Eastern staff in Swedish homes or Spanish-speaking providers for Latino families, it can be observed that formal caregivers try to adjust to the diverse backgrounds of the residents and families (Martinez et al., 2022; Antelius and Plejert, 2016). However, these efforts usually depend on the caregiver’s personal dedication rather than support from the system itself. Because of this, families often feel they are filling in the gaps, doing their best to make sure their loved ones are understood and cared for. Therefore, the choice of care setting and the responsibility for caregiving are not only shaped by individual choices but also structural and contextual factors such as culture, class, generation, age, language and migration history (Martinez et al., 2022; Tezcan-Güntekin, 2018).

From a structural intersectionality lens, we interpret care preferences and expectations not solely as expressions of personal choice, but as products of broader power relations and systems of oppression (Homan et al., 2021). As it can be seen in the example of filial piety, the expectation that female adult children care for older family members may reflect not only intergenerational values but also internalized social norms about gender and family responsibility. Similarly, when individuals living with dementia express a preference to receive care from female relatives, these preferences are under the influence of longstanding social norms about caregiving roles. Especially in the migration context, gendered care is connected to the maintaining familial and cultural identity (NÆSs and Moen, 2014). Our findings reflect a rather traditional understanding that caregiving is not just a familial responsibility but also that it is performed mostly by women (Martinez et al., 2022). However, this phenomenon differs when acculturation, generation, education and financial factors are involved in to the picture (Czapka and Sagbakken, 2020). Within our review, we observed that despite the prevalence of gendered care, individual agency remained significant in how preferences were practiced and negotiated within families.

One of the challenges in the expression of preferences is that post-migrants and ethnic minority groups with dementia and their informal care providers do not have knowledge about dementia or information about culturally appropriate dementia services (Chejor et al., 2022; Lewis et al., 2021). With regard to barriers and language issues in the healthcare system, this lack of information suggests that detecting preferences, needs or expectations from an intersectional perspective may be the next step. Cultural and religious values such as familism, collectivism, and emotionalism play a significant role in shaping attitudes toward caregiving (Hossain and Khan, 2020; Dilworth-Anderson and Gibson, 2002). These norms often discourage individuals from expressing preferences that conflict with traditional expectations. For example, concepts such as karma, beliefs such as “if it comes from God, we should not act against God” or an understanding of dementia as divine punishment can prevent individuals from seeking external care or discussing their needs openly (Hossain and Khan, 2020; Czapka and Sagbakken, 2020; Sagbakken et al., 2018b; Taiebine, 2024). Another reason for the inability to express preferences is the perception of dementia as a “normal” outcome of the aging process (NÆSs and Moen, 2014). These perspectives may be obstacles to the timely diagnosis and management of dementia symptoms (Kovaleva et al., 2021). However, this perception cannot be understood only with respect to culture; rather, it involves interlinked determinants such as education, traditional norms, economic factors and religious values (Kovaleva et al., 2021; NÆSs and Moen, 2014; Sagbakken et al., 2018b). Ethnographic study conducted with Jewish community shows that the social inclusion of people with dementia is connected to cultural and religious belief about taking care of each other, helping them to access to a comprehensive network of social and health institutions (Motta-Ochoa et al., 2021). As Martinez et al. (2022) suggested, educating families about available support services and integrating culturally and religiously sensitive care into daycare facilities or nursing homes may play a crucial role in helping people make informed choices.

### 4.1 Individual and institutional barriers, facilitators, and recommendation

An intersectional perspective is important for questioning power dynamics and inequalities in society. It is also significant to recognize the gap in research, practice and social mindsets spanning from the individual to institutional to societal level. The underrepresentation of post-migrants and ethnic minority groups with dementia in research combined with the lack of care options indicates the challenges and barriers in the health system (Roes et al., 2022). That the healthcare system is not inclusive enough creates these challenges, including a lack of culturally sensitive dementia services, language barriers, and non-existing care concepts addressing diversity as an important aspect in professional care (Czapka and Sagbakken, 2020; Nkimbeng et al., 2022; Sagbakken et al., 2018b). Reports from the studies indicate that discrimination, stereotyping, and feeling dismissed by healthcare professionals further create mistrust and fear, discouraging post-migrants and ethnic minority groups from accessing care. Language difficulties, especially compounded by dementia-related loss of second-language skills, further complicate communication of needs and preferences (Rosendahl et al., 2016; Sagbakken et al., 2018a). As Czapka and Sagbakken (2020) note, structural and institutional factors shape the conditions in which individuals make decisions and influence what choices are available to them.

In order to decrease the burden, researchers should consider PCC together with intersectionality to understand the complex experiences of minority groups. Larger-scale studies are required to better understand how language, identity and communication practices form the experiences of people with dementia (Plejert et al., 2017). Additionally, shifting the research focus from deficits toward competencies and potential for social engagement can lead to more empowering care practices and informed policy development (Plejert et al., 2017). Regarding practice, adapting care routines to honor these preferences by recognizing the unheard diverse needs can improve comfort, reduce distress, and promote self-esteem in care settings (Fazio et al., 2018). Instead of standardized services, failing to embrace cultural and linguistic needs and leading to further marginalization, inclusive care services enhances the sense of belonging and dignity of people with dementia (Motta-Ochoa et al., 2021).

Furthermore, the representativeness of care staff who share a cultural background can significantly enhance the well-being of residents (Sagbakken et al., 2020). Our research indicates that cultural, linguistic, and systemic barriers that hinder trust, communication, and quality of care often strain the dyadic relationship between persons with dementia and formal caregivers. Across various contexts, from Alaska to Sweden to Latino and Korean communities, language barriers and lack of cultural competence emerge as central challenges, leading to unmet needs, misinterpretation of behaviors, and emotional distress for both people with dementia and caregivers. Employing healthcare professionals from similar linguistic or cultural backgrounds can enhance trust and communication (Schouten et al., 2020). Building intercultural competence in healthcare setting is key to improving care for post-migrants and ethnic minority groups with dementia. Training healthcare workers by improving their understanding of culturally shaped behavior and helping them feeling more confident in cross-cultural situations can enhance the communications with the people with dementia (Beck et al., 2024; Lindtner-Rudolph et al., 2025). They should also prioritize the adoption of PCC approaches with the trainings and workshops deepening the diverse needs of vulnerable groups (Taiebine et al., 2025).

For stakeholders, it shows a commitment to fair and respectful care by making intercultural awareness a part of everyday practice (Lindtner-Rudolph et al., 2025). Enabling individuals to express their preferences carries a great importance in enhancing dialogue, negotiation and knowledge exchange. This allows underrepresented groups to be recognized and fulfilled their needs in the health care system (Holman et al., 2021). To develop responsive and equitable policies, it is essential to consider how the interacting factors such as culture, religion, age, gender, education, health literacy, and language competencies shape specific preferences and how best to adapt to these requirements (Czapka and Sagbakken, 2020; Homan et al., 2021; Leszko and Allen, 2024; Taiebine et al., 2025; Holman et al., 2021). In addition, making intercultural training a requirement for health care professionals may help to reduce health inequalities and creates more inclusive environments (Lindtner-Rudolph et al., 2025). Healthcare systems need to promote inclusive policies considering the diversity of our societies (Schouten et al., 2020).

## 5 Strengths and limitations

We implemented a comprehensive search strategy across multiple literature databases and conducted forward and backward citation tracking to identify as many relevant studies as possible. To our knowledge, no qualitative scoping review has examined this topic. One of the strengths of this review is that the studies were from several countries and represented various communities, which allowed us to collect information about underrepresented groups. The studies were also conducted in a variety of settings and capture various aspects of preferences, which is an important point. However, this diversity also limits the generalizability of some findings.

We derived our search terms according to the research questions by conducting an initial review of the literature supplemented with broader terms. However, this approach may have excluded some relevant studies. For example, filial piety emerged as a relevant concept and incorporating the term as a keyword would help contribute the future studies. Furthermore, our research adopted an interpretative approach to examine preferences. While this study incorporated a range of articles, we identified additional literature that explored the experiences of individuals with dementia by considering the heterogeneity and diversity in the group. However, the primary focus of these studies was the burden of caregiving. Since our focus was the experiences, wishes, needs and expectations of this group with the aim of emphasizing empowerment and autonomy, we excluded such studies. From an alternative interpretative perspective, these studies may provide valuable insights into the interpretation of needs by examining the challenges this population faces within the health system.

Adopting an intersectional perspective helps us to understand how power works in differentiated way by shaping and utilizing overlapping identity categories. We focused on several overlapping categories that leads in some cases production of similar results between the study populations and excluded population group. For example, hesitation of undress in front of healthcare professionals may be closely linked to the intersection of cultural values, religious belief and gender; still, a white male resident in nursing home may have similar experiences of shame when exposed in front of others. Similarly, bringing a conclusion that a population group embracing the same categories of migration, gender, age or religion would always have the similar preferences can be also mistaken. However, considering these categories and identities with an intersectional perspective can spark further inquiry related to the interplay of multiple social dynamics and power relations. The future of intersectionality studies depends on how well scholars reveal intersecting power and inequality while critically examining the tools and concepts shaping the knowledge (Cho et al., 2013).

## 6 Conclusion

This review indicate the importance of integrating a more intersectional approach to understanding the wishes, needs, and preferences of post-migrants and minority groups with dementia and their caregivers. In contrast to studies that focus only on cultural sensitivity, this study highlights the need to provide tailored services for the intersecting experiences of post-migrants and ethnic minority groups with dementia and their caregivers. To close this gap, standardized procedures that are adapted to the multilayered preferences of this group, both in home care and in nursing homes, are needed. Addressing these multifaceted needs can promote inclusivity, improve health outcomes, and increase the quality of life of individuals with dementia and their caregivers in diverse communities.

## Data Availability

The original contributions presented in the study are included in the article/Supplementary material, further inquiries can be directed to the corresponding author.
