# Supplementary material for: Intersectional research on dementia care for post-migrants and ethnic minority groups: a scoping review
Source: Front Dement. 2025 Aug 18;4:1596395. doi: 10.3389/frdem.2025.1596395 (PMC12399571; doi:10.3389/frdem.2025.1596395)
Supplement: Supplementary file 1 [file Data_Sheet_1.pdf]

Supplement I: Data Extraction Table

Author Details, Year, Institution, and Country	Study Characteristics (Study design, language, and publication type)	Aim	Population and Sample Size of CALD	Information about CALD	Setting	Analysis through the lens of intersectionality	Analysis of Preferences	Results/Take-away Message
**[1] Antelius et al. (2016)** Dementia caregiving targeted towards Middle Eastern immigrants living in Sweden, Linköping University, Sweden	In-depth interviews<br><br>English, peer-reviewed article	Exploring the perspectives of dementia as a culturally and socially shaped illness in order to clarify such perceptions and experience in regard to ethno-culturally profiled dementia care in Sweden	N= 10 care staff	Middle Eastern immigrants in Sweden	A residential dementia care facility with Arab focus, a residential dementia care facility with (so-called) Oriental focus, and a day center for persons with dementia who have Middle Eastern backgrounds	The intersection of culture, generation and migration	Preferences in the care setting	There is a strong cultural expectation to care for older age family members at home. Sending them to nursing homes is often seen as unusual, and doing so can lead to feelings of guilt and shame. These cultural values emphasize familial responsibility, and people may face social stigma or judgment if they use for formal care, as it is perceived as abandoning or neglecting their loved ones.
**[2] Chaouni et al. (2024)** The influence of religion on the care experiences of family carers of older migrants with dementia in Belgian cities,	In-depth interviews<br><br>English, peer-reviewed article	Exploring the influence of religion on the caregiving experiences of family caregivers responsible for older labor	N= 34 family caregivers	Migrant group with Moroccan, Turkish, and Italian descent with dementia in Belgium	Community setting	The intersection of age, religion, and migration	Other daily preferences connected to religious practices	Religious practices, such as prayer and attending mosque or church, provided comfort and emotional relief for both caregivers and older post-post-migrants with dementia. These shared religious activities brought joy and strengthened bonds, making them beneficial

Vrije Universiteit, Belgium		migrants residing in Belgian cities						for both the caregiver and the older age person.<br>A lack of cultural sensitivity in care caused distress for both the older age patient and the family, as staff refused to accommodate the religious preferences despite explanations.
						The intersection of age, culture, religion, and generation	Preferences over informal care provider	Caregiving for older age relatives with dementia is driven by cultural and religious values. Respondents, especially those of Turkish and Moroccan backgrounds, saw it as a natural duty, often citing Islamic teachings about respect, reciprocity, and care for parents as key motivations.
						The intersection of culture and religion	Preferences in the care setting	Respect for the religious identity and practices of older post-migrants with dementia is crucial in determining whether families will use professional care. Both religious and non-religious family caregivers valued professional caregivers who respected religious customs, such as halal food, prayer, and religious attire.

Supplement I: Data Extraction Table

						The intersection of religion and migration	Expectations/ Preferences in nursing homes/care institutions	Muslim caregivers find it challenging to find religion-sensitive care for older age relatives with dementia, as Belgium lacks Muslim-inspired care facilities. While Christian-inspired care can meet the needs of Italian post-migrants, there is a gap in care options for Muslim families.
**[3] Czapka et al. (2020)** "It is always me against the Norwegian system." barriers and facilitators in accessing and using dementia care by minority ethnic groups in Norway: a qualitative study, Oslo Metropolitan University, Norway	Semi-structured in-depth interviews<br><br>English, peer-reviewed article	Examining the obstacles and facilitators in accessing and using dementia care services by minority ethnic groups in Norway	N= 8 families<br><br>N= 5 key representatives of immigrant communities<br><br>N= 6 representatives of health and care personnel	Participants from Somalia, Poland, Croatia, Pakistan, India, Turkey and one of the islands in the Atlantic Ocean	Nursing homes, memory clinics, day centers, home care services, and community setting	The intersection of age, culture, generation, and migration<br><br>The intersection of culture, generation, and migration	Preferences in the care setting<br><br>Preferences in the care setting	Cultural obligations to care for older age family members significantly restrict access to dementia care services, as many caregivers view this responsibility as a moral duty rather than a burden. The norm of reciprocity, rooted in love and familial responsibility, leads to reluctance in considering nursing homes or home-based services, with strong family ties influencing caregiving practices within minority communities.<br><br>While first-generation caregivers may feel compelled to uphold cultural values, second-generation individuals are more open to institutional care, reflecting a desire for less responsibility and greater support from health authorities. This creates tension between the

								familialistic caregiving practices and the more defamilialized approach promoted by Norwegian society.
						The intersection of tradition and migration	Food preferences	Dietary habits and preferences are heavily influenced by cultural backgrounds, and a lack of cultural sensitivity in care settings can lead to feelings of disrespect among minority groups. Participants expressed the need for better communication and inclusivity in care services.
						The intersection of culture, religion and migration	Expectations in nursing homes/care institutions	The limited opportunities for religious practices in Norwegian care institutions are a significant concern for some families, leading them to hesitate in placing relatives with dementia in such facilities. Participants noted that infrequent visits from religious leaders, such as a priest visiting only once a week, do not meet the needs of patients who desire more frequent access to spiritual support.
**[4] Dilworth-Anderson et al. (2002)** The Cultural Influence	Secondary data from structured	Examining how cultural values, norms, and beliefs	N=121 caregivers	Caregivers, self-identified as	Community setting involving home-care programs,	The intersection of age, cultural	Preferences connected to informal care provider	Chinese American caregivers express the expectation of other family members, mostly young

of Values, Norms, Meanings, and Perceptions in Understanding Dementia in Ethnic Minorities, The University of North Carolina at Greensboro, USA	and semi-structured interviews<br><br>English, peer-reviewed article	form the meanings diverse ethnic groups assign to dementia		being of African, Chinese, European, and Hispanic in the US	general healthcare setting, adult day health centers etc.	values, and generation		generation, to provide care and do not seek medical treatment.
**[5] Hossain et al. (2020)** Barriers to access and ways to improve dementia services for a minority ethnic group in England, Keele University, the UK	Phase (1) Focus group interview; Phase (2) Semi-structured interview<br><br>English, peer-reviewed article	Examining the barriers to health care service use in the Bangladeshi community living in the United Kingdom	Phase (1) N=21 general members of the Bangladeshi community<br><br>Phase (2) N= 6 Bangladeshi family caregivers	Bangladeshi people in UK	Community setting	The intersection of culture and migration<br><br>The intersection of culture, gender, and religion,	Preferences connected to informal care provider<br><br>Preferences connected to informal care provider	Bangladeshi community prioritizes family as primary caregivers, which can create barriers to seeking external dementia care.<br><br>Some family caregivers, particularly in the Bangladeshi Muslim community, may lack family support and face cultural constraints that complicate caregiving. One male caregiver had to pay for additional culturally appropriate services to manage his father's incontinence. Despite receiving support from a care agency, it was insufficient for his needs, highlighting the gaps in culturally sensitive care options and the financial burden on caregivers.

Supplement I: Data Extraction Table

						The intersection of culture, religion and migration	Preferences in the care setting	There is a strong belief that family should be the primary caregivers for people with dementia, and care should ideally be provided within the family home. In addition, cultural and religious beliefs significantly influence Bangladeshi Muslim family caregivers' reluctance to utilize residential care homes for relatives with dementia. Participants expressed a strong preference for home-based care, citing concerns about adherence to Islamic practices, such as dietary restrictions (halal food) and cleanliness, as well as doubts about care home staff's ability to meet these needs.
						The intersection of culture, religion and gender	Preferences in the care setting	Caregivers prioritize gender over religion and ethnicity when selecting respite carers, indicating a preference for Bangladeshi same-sex Muslim carers first, followed by non-Muslim Bangladeshi carers, and non-Bangladeshi Muslim carers.
						The intersection	Other daily preferences	Personal hygiene is a crucial aspect for religious Bangladeshi Muslims, with family caregivers

Supplement I: Data Extraction Table

						of culture and religion<br><br>The intersection of culture and religion	connected to hygiene<br><br>Expectations in nursing homes/care institutions	emphasizing the importance of adhering to strict hygienic rules in caregiving practices.<br><br>Family caregivers emphasized the importance of language, suggesting that care workers learn Bengali to better engage with clients and their families. Such initiatives would help ensure that caregivers feel their cultural and religious backgrounds are recognized and valued within mainstream healthcare services.
**[6] Im et al. (2022)** Attitudes toward Alzheimer's disease and dementia caregiving and health outcomes: Racial and ethnic differences, Nell Hodgson Woodruff School of Nursing, Emory University, USA	Cross-sectional online survey<br><br>English, peer-reviewed article	Discovering racial/ethnic variances in the attitudes toward Alzheimer's disease and dementia caregiving	N = 172 family caregivers of persons living with AD	African Americans, Hispanics, and Asian groups	Online communities and groups	The intersection of age, ethnicity, gender, and race	Preferences connected to informal care provider	Asian midlife women caregivers tends not to share responsibilities, rather centralize the caregiving to serve care recipient because of understanding of filial piety.

Supplement I: Data Extraction Table

**[7] Kong et al. (2010)** The Experience of Family Caregivers of Older Korean Americans With Dementia Symptoms, University of Maryland, USA	Semi-structured focus group interview<br><br>English, peer-reviewed article	Examining the experience of dementia caregiving among Korean Americans	N= 23 family caregivers	Korean Americans in the US	Home care, adult day care, respite care, and nursing home care	The intersection of culture and generation	Preferences connected to informal care provider	The "Korean way of thinking," which emphasizes family and filial piety, significantly influences caregiving experiences among Korean post-migrants, particularly regarding nursing home placement.
						The intersection of age, culture, generation, and migration	Preferences connected to informal care provider	Korean post-migrants in the United States maintain a complex relationship with their cultural identities, blending traditional Korean values rooted in Confucianism with elements of American culture. While some informants reported limited changes in their fundamental ways of thinking after immigration, others noted that recent post-migrants appear more Americanized. Those who immigrated earlier often retain traditional values, even as their lifestyles may adopt American practices in areas such as eating, dressing, and housing.
						The intersection of age, culture, gender, and generation	Preferences connected to informal care provider	Despite changes in their life circumstances, such as increased workforce participation among women and diminished extended family support after immigration, individuals within

								the Korean community still feel pressured to uphold traditional values of filial piety.
						The intersection of culture, generation and gender	Preferences in the care setting	Informants within the Korean community strongly associate filial piety with the obligation to care for aging parents at home, viewing nursing home placements as a failure to fulfill this duty. Many expressed guilt and self-consciousness regarding these decisions. The negative perceptions of nursing homes, compounded by the stigma associated with not adhering to traditional values, deeply affect caregivers' feelings and choices, highlighting the enduring impact of cultural beliefs on caregiving practices.
						The intersection of culture and migration	Other daily preferences in eating, dressing, and housing	The adoption of Americanized lifestyle while maintaining traditional Confucian values creates a generational divide where older post-migrants feel caught between maintaining traditional beliefs and adapting to the realities of modern immigrant life, including increased workforce

Supplement I: Data Extraction Table

								participation and diminished extended family support.
						The intersection of culture, language and migration	Expectations in nursing homes/care institutions	Language barriers significantly hinder communication between Korean residents and nursing home staff, leading to negative experiences for both the residents and their families. Many caregivers emphasized the need for bilingual Korean American staff to improve communication, ensure comfort, and better accommodate the cultural needs of Korean residents with dementia. This highlights the critical role that effective communication plays in the caregiving experience within nursing homes.
						The intersection of culture and gender	Expectations in nursing homes/care institutions	Cultural norms surrounding modesty, particularly in traditional Korean culture, can lead to resistance and aggressive behaviors in individuals with dementia during personal care activities, highlighting the importance of culturally sensitive approaches by caregivers.

Supplement I: Data Extraction Table

						The intersection of culture, gender, and migration	Other daily preferences of bathing	Cultural preferences, such as the discomfort with nudity during bathing among Korean women, can significantly impact the daily care experiences of individuals with dementia, leading to misunderstandings and challenging behavior, when healthcare professionals do not consider these values.
						The intersection of culture and migration	Other daily preferences connected to sleeping habits	In Korean culture, daily preferences, such as sleeping on the floor instead of a bed, reflect traditional practices and habits that individuals with dementia may continue to prefer.
**[8] Lawrence et al. (2011)** Threat to Valued Elements of Life: The Experience of Dementia Across Three Ethnic Groups, King's College London, the UK	In-depth interviews<br><br>English, peer-reviewed article	Examining the perceived reality of people living with dementia within the 3 largest ethnic groups in the United Kingdom	N= 30 people with dementia	Black Caribbean and South Asian people with dementia in UK	Multidisciplinary health and social care, memory clinics, day centers, community programs, and dementia-specific day center and group	The intersection of age, culture, generation, and migration	Preferences connected to informal care provider	South Asian participants valued family support but did not see it as extraordinary, viewing it as a reciprocal relationship where children give back for the care they received. Concerns about burdening family were balanced by a sense of having fulfilled parental duties, with many expecting ongoing family care.

Supplement I: Data Extraction Table

**[9] Lee Casado et al. (2015)** The Experience of Family Caregivers of Older Korean Americans With Dementia Symptoms, University of Maryland, USA	Focus group interviews<br><br>English, peer-reviewed article	Examining the experience of dementia caregiving among Korean Americans	N= 23 family caregivers	Korean Americans in the US	Home care, community healthcare settings, and support group environments	The intersection of culture, gender, and migration	Preferences connected to informal care provider	The expectation in traditional Asian/Korean culture for women, particularly wives and daughters-in-law, to assume caregiver roles for family members. This expectation creates a cycle of inherited responsibilities, where caregivers feel obligated to fulfill the same duties as previous generations, reinforcing gender-assigned roles within family dynamics.
						The intersection of age, culture, generation, and migration	Preferences connected to informal care provider	The strong cultural tradition of familism among Korean American caregivers fosters a deep sense of responsibility and commitment to caregiving, often leading them to shoulder the burden independently without seeking external support. This adherence to culturally expected roles can create family tensions, particularly between adult children and their spouses.
**[10] Leszko et al. (2024)** Caring From a Distance: Experiences of Polish Immigrants in the United States Providing Care to Parents With	Semi-structured interviews<br><br>English, peer-reviewed article	Examining the experiences of adult children and immigrant caregivers of a parent with dementia locating in Poland.	N= 37 caregivers	Polish Immigrants in the US	Transnational caregiving	The intersection of age, culture, generation, and migration	Preferences connected to informal care provider	In Polish culture, there is a strong sense of filial obligation and solidarity regarding the care of older age parents. Participants emphasized that caring for parents is seen as a duty and a way to show appreciation and respect, reflecting a tradition of

Supplement I: Data Extraction Table

Dementia Overseas, University of Szczecin, Szczecin, Poland						The intersection of class, culture, and migration	Preferences in the care setting	reciprocity in family care that is not viewed as a burden.<br><br>Caregivers struggle with the decision to place a person with dementia in a nursing home due to financial constraints, family values, and fears of being perceived as unloving..
**[11] Lewis et al. (2021)** "Making Sense of a Disease That Makes No Sense": Understanding Alzheimer's Disease and Related Disorders Among Caregivers and Providers Within Alaska Native Communities, University of Minnesota Medical School, USA	Semi-structured interview<br><br>English, peer-reviewed article	Understanding Alzheimer's disease and related disorders among caregivers and providers within Alaska native communities	N = 21 caregivers<br><br>N = 15 health care providers	Caregivers and Providers Within Alaska Native Communities	Urban centers and rural communities	The intersection of ethnicity, culture, and spirituality	Other daily preferences – Perceived treatment methods	The use of traditional healing/medicines and the preferences for holistic approaches in treatment of the disease is common in Alaska Indian/Alaska Natives.

**[12] Liu et al. (2008)** Re-examining the relationships among dementia, stigma, and aging in immigrant Chinese and Vietnamese family caregivers, UC Davis Medical School, USA	In-depth interviews<br><br>English, peer-reviewed article	Understanding the relationship of stigma and dementia in Chinese and Vietnamese communities and comparing the meanings families assign to dementing illness and help-seeking responses	N= 32 family caregivers	Chinese and Vietnamese group in the US	Primary care, home health agencies, adult day health centers, and nursing homes	The intersection of age, culture, generation, and migration	Preferences connected to informal care provider	In Chinese culture, care for the older age is seen as a reciprocal "parent-child contract" rooted in Confucian values, where children, especially the eldest son, provide support in exchange for services from the older age, maintaining balance between generations.
**[13] Martinez et al. (2022)** The Experience of Alzheimer's Disease Family Caregivers in a Latino Community: Expectations and Incongruences in Support Services, California State University Long Beach, USA	In-depth interviews<br><br>English, peer-reviewed article	Examining the care experiences of Latino family caregivers to persons with Alzheimer's disease and related dementias (ADRD), and understanding the cultural influences between provider perspectives with the expectations of	N = 24 caregivers N=10 service providers	Latino Community in the US	Hospital-based services and local agencies	The intersection of education, culture and migration<br><br>The effect of cultural values, gender, and migration	Preferences in the care setting<br><br>Preferences connected to informal care provider	There is a preference over home-care because many service users are not educated enough to know what daycare can do for them.<br>Low levels of service utilization have been associated with familismo and personalismo.<br>The formal care provided by institutions does not seems as alternative, but rather they reach out the family for help.<br><br>Caregiving responsibilities are mostly attached with women.

		Latino ADRD caregivers				The intersection of age, cultural values, and generation<br><br>The effect of financial constructio n, class and culture	Preferences connected to informal care provider<br><br>Preferences connected to informal care provider	Providing care to the older age is expressed as a certain duty, which is culturally constructed within the framework of maternal love, sacrifices, and sense of control. This put family as the center of caregiving.<br><br>Although the preferences of care provided for family is dominant, it can be also shaped by financial necessity.
**[14] Næss et al. (2014)** Dementia and migration: Pakistani immigrants in the Norwegian welfare state, Oslo and Akershus University College of Applied Sciences, Norway	Field observations and in-depth interviews<br><br>English, peer-reviewed article	Understanding how Norwegian-Pakistani families navigate dementia between their culturally defined care practices and the Norwegian health system, which emphasizes public care and biomedical intervention	N= 22 families and healthcare employees	Norwegian-Pakistanis in Norway	Clinical setting, home care, and community setting	The intersection of beliefs, culture, and migration<br><br>The intersection of age, culture, generation, and migration<br><br>The intersection of age,	Other daily preferences including coping approaches<br><br>Preferences connected to informal care provider<br><br>Preferences in the care setting	While Norwegian-Pakistanis are not entirely opposed to allopathic medicine, there is some uncertainty about replacing or integrating biomedical understandings of dementia with traditional approaches.<br><br>Unlike ethnic Norwegians who typically rely on public care for dementia, Norwegian-Pakistanis are less likely to seek public help due to the cultural emphasis on and family caregiving.<br><br>In the Norwegian-Pakistani community, strong cultural values around filial duty and

Supplement I: Data Extraction Table

						culture, generation, religion and migration		family reputation create barriers to seeking public care. Caregiving is seen as both a moral and emotional responsibility, with concerns about community judgment, cultural insensitivity in public institutions, and maintaining the dignity of elders driving a preference for family-based care.
						The intersection of culture, gender, generation, religion and migration	Preferences in the care setting	The Norwegian-Pakistani community, emphasizing collective effort and individual sacrifice, consider public care a secondary or last resort option.
**[15] Nkimbeng et al. (2022)** The Immigrant Memory Collaborative: A Community–University Partnership to Assess African Immigrant Families' Experiences with Dementia, University of Minnesota School	Phase (1) Community-Based Participatory Research; Phase (2) semi-structured focus group interview; Phase (3) survey<br><br>English, peer-	Exploring dementia care needs and resources in the African immigrant	Phase (1) N=20 community stakeholders for the participatory research<br><br>Phase (2) N=24 African immigrants for community	African Immigrant in the US	Community setting with family, nursing home, and institutional care	The intersection of culture and migration	Preferences in the care setting	Participants preferred in-home care from family due to cultural values, while also noting the stigma surrounding dementia that leads individuals to avoid diagnosis for fear of being treated differently.

of Public Health, USA	reviewed article		conversation<br><br>Phase (3) N= 170 (ongoing)					
**[16] Ramsay et al. (2017)** Migrant caregiving for family members with mild cognitive impairment: an ethnographic study, The University of Melbourne, Australia	In-depth interviews with ethnographic approach<br><br>English, peer-reviewed article	Examining the cultural wishes, needs, beliefs and health seeking behaviors of migrant Turkish family member caregivers	N= 10 caregivers	Turkish caregivers in Australia	General practice clinic and home care setting	The intersection of culture, generation and migration<br><br>The intersection of age, culture, religion, and migration	Preferences in the care setting<br><br>Preferences in the care setting	Caregivers often feel they don't need formal healthcare services due to strong family support rooted in Turkish and Islamic culture. They believe it is their duty to care for their parents out of respect, and many view placing an elder in a care facility as a sin, as it fails to repay the sacrifices their parents made for them. Some caregivers worry that their family members would be devastated to know they were being placed in a nursing home, viewing it as a death sentence.<br><br>People feel it would be a sin to not care for their parents, as they believe they can never repay the love and support their parents gave them while raising them.

Supplement I: Data Extraction Table

						The intersection of culture, gender, and migration<br><br>The intersection of age, culture, generation and migration	Preferences connected to informal care provider<br><br>Preferences connected to informal care provider	Caregiving daughters in Turkish culture face increased stress due to cultural expectations that place greater familial responsibilities on women, who are seen as primary caregivers.<br><br>Closer age between generations leads to larger family networks and greater responsibilities for extended family members, including dependent children.
**[17] Richardson et al. (2019)** At the intersection of culture: Ethnically diverse dementia caregivers' service use, Ohio State University, USA	In-depth interviews<br><br>English, peer-reviewed article	Exploring cultural factors influencing ethnically diverse dementia caregivers' experiences and use of services	N= 15 caregivers	Hispanic, African American, and South Korean participants	Community setting	The intersection of age, culture, generation, gender, and migration<br><br>The intersection of culture, generation, religion, and migration	Preferences connected to informal care provider<br><br>Preferences in the care setting	Gender role conflicts arise among Korean American caregivers, with daughters providing hands-on care and sons offering financial support, while Hispanic caregivers feel a strong obligation to care for loved ones at home, reflecting cultural values around filial care.<br><br>Cultural traditions significantly influence caregiving perspectives among Hispanic and Korean families. Hispanic caregivers emphasize the importance of maintaining family care, viewing nursing homes negatively and as places to die, while expressing a strong

Supplement I: Data Extraction Table

								commitment to caring for parents at home. Similarly, a Korean caregiver acknowledges that nursing homes are not commonly accepted in their culture.
						The intersection of culture and religion	Other daily preferences	Cultural traditions, such as using candles, remain important for Hispanic caregivers, when their family members cope with dementia.
						The intersection of tradition and migration	Food preferences	Some Korean families found nursing homes lacking in cultural sensitivity, particularly regarding food preferences.
						The intersection of culture and language	Expectations in nursing homes/care institutions	Language barriers limit service access for Hispanic and Korean service users. Religion and spirituality play important roles for African American caregivers. However, service providers typically operate in a secular context that is disconnected from religious-based services.

Supplement I: Data Extraction Table

**[18] Sagbakken et al. (2018)** Dementia and Migration: Family Care Patterns Merging With Public Care Services, The Norwegian Centre for Migration and Minority Health (NAKMI), Norway	In-depth interviews and dyad interviews<br><br>English, peer-reviewed article	Understanding the perspectives and experiences of family members and professional caregivers regarding the care provided to immigrants with dementia or age-related cognitive impairment	N= 12 relatives<br><br>N=18 health personnel	Immigrants from Pakistan, India, Afghanistan, Iran, Turkey, Algeria, Mexico, Chile, Poland, and Bosnia in Norway	Community-based home care, nursing homes, day care centers, geriatric polyclinic, psychiatric polyclinic, "memory clinics", community health center	The intersection of age, culture, generation, gender and migration	Preferences connected to informal care provider	Cultural norms surrounding filial piety and caregiving create a sense of obligation for family members to care for their older age relatives, particularly among women. However, these expectations are complicated by factors such as migration, gender roles, and the challenges of balancing caregiving with personal responsibilities. Many caregivers experience stress and ambivalence as they navigate these pressures, leading to a limited understanding from non-caregivers about the difficulties faced by those providing care. The stigma associated with using nursing homes further compounds the challenges, reflecting a deep-rooted cultural belief in the importance of family care.
**[19] Sagbakken et al. (2020)** How to adapt caring services to migration driven diversity? A qualitative study exploring challenges and possible adjustments in the	Individual and dyad interviews, and focus groups discussions<br><br>English, peer-reviewed article	Developing more knowledge of challenges and potential changes related to receive and provide public care for people living with dementia with	N=19 single interviews, N=3 dyad interviews, and N=16 focus groups with older immigrants, relatives of immigrants	Older immigrants and the relatives/family members from culturally and linguistically various background (Pakistan,	GP centres, nursing homes, day-care centres, home-based services, geriatric and psychiatric polyclinics, and hospital-based memory clinics	The intersection of culture and religion<br><br>The intersection of religion	Preferences in the care setting<br><br>Other daily preferences in care	Culturally or religiously influenced family care preferences make it hard to adapt to the standardized care in nursing homes or day care centers.<br><br>Some relatives, influenced by religious beliefs, felt that all efforts, including providing nutrition, should be used to

care of people living with dementia, Oslo Metropolitan University, Norway		an immigrant or minority ethnic background	with dementia, and health personnel	India, Afghanistan, Iran, Turkey, Algeria, Mexico, Chile, Poland, and Bosnia, China, Vietnam, Lebanon, and Sri Lanka) in Norway		and migration<br><br>The intersection of culture, religion, and migration<br><br>The intersection of culture and language, and migration	 <br>Food preferences<br><br>Expectations in nursing homes/care institutions	preserve life until God decides the time of death, which sometimes contrasts with the medical approach to end-of-life care.<br><br>Food habits, especially access to culturally appropriate and varied meals, are important for older post-migrants in care facilities.<br><br>Older post-migrants with dementia and their families expressed a desire for more staff from the residents' countries of origin to improve communication and cultural understanding. Some expressed that the residents with dementia should have the right to interact with staff who understand their language and cultural background.
**[20] Shanley et al. (2012)** A qualitative study into the use of formal services for dementia by carers from culturally and	Focus group interviews, one-to-one interviews<br><br>English, peer-reviewed article	Examining the experiences and understanding of dementia caregiving practices of people from	N= 121 family carers,<br><br>N= 60 health professionals	Italian, Chinese, Spanish and Arabic-speaking communities in southwestern	In-home personal care, in-home domestic help, in-home and centre-based respite, residential	The intersection of culture, language, and migration	Expectations in nursing homes/care institutions	Family carers prefer professional caregivers supporting the homecare services to share their language and cultural background, as bilingual workers can provide more personalized and culturally tailored care.

linguistically diverse (CALD) communities, University of New South Wales, Australia		CALD backgrounds		Sydney, Australia	care, and community services	The intersection of culture, gender, and religion	Expectations in nursing homes/care institutions	Cultural and religious beliefs significantly impact the acceptability of home care services. Italians found the cleaning standards inadequate, while Arabic communities were uncomfortable with outsiders and certain personal care practices, especially across genders, due to religious and cultural values.
						The intersection of class, culture, and migration	Expectations in nursing homes/care institutions	Cultural perceptions influence the use of services in culturally and linguistically diverse communities. They may be unfamiliar with available services, leading to reluctance in seeking help. For example, in Chinese culture, receiving community services may be associated with poverty and lack of family support, making people hesitant to use them.
						The intersection of tradition and migration	Food preferences	The lack of culturally familiar food is a barrier for culturally and linguistically diverse communities in using mainstream services.

Supplement I: Data Extraction Table

						The intersection of culture, generation, and migration	Preferences connected to informal care provider	Cultural traditions, such as filial piety in Chinese and Arabic communities and "familismo" in Italian and Spanish cultures, significantly influence the use of services, with a strong expectation for family to care for older age members. Feelings of guilt or pressure to provide home care are common, though some openness to residential care is emerging due to Western influences and acculturation, especially in families that have been in Australia longer.
**[21] Tezcan-Güntekin (2018)** Stärkung der Selbstmanagement-Kompetenzen pflegender Angehöriger türkeistämmiger Menschen mit Demenz, Bielefeld University, Germany	Expert interviews<br><br>German, dissertation	Investigating the state of family caregivers of Turkish-origin individuals with dementia with a focus on their stressors, resources, and needs	N=11 experts in the medical/social work/nursing fields, N=12 family caregivers	Turkey-origin people in Germany	Home care setting and outpatient care services	The intersection of age, culture, generation, gender, and migration<br><br>The intersection of culture, gender, and migration	Preferences connected to informal care provider<br><br>Preferences connected to informal care provider	Caregiving roles are often shaped by traditional gender norms, with women typically providing direct care and men handling organizational tasks. Conflicts may arise due to differing family expectations.<br><br>Caregiving is often perceived as a female responsibility, particularly in families with patriarchal structures. While women are typically the primary caregivers, men also face challenges when involved in care. Although institutional care is generally stigmatized, open family discussions can

								sometimes lead to its consideration as a viable option.
						The intersection of class, culture, and migration	Preferences in the care setting	Financial barriers significantly influence caregiving decisions, with many families choosing for exclusively familial care due to the cost of professional services like day care or nursing homes. Misunderstandings about care allowances discourage the use of home care services.
						The intersection of culture, religion, and migration	Preferences in the care setting	Societal pressure within Turkish communities strongly influences caregiving decisions, with a cultural expectation that families provide care themselves and a stigma against seeking professional support. This pressure often delays the use of external care, increasing stress on family caregivers. The taboo around discussing dementia and fear of judgment further discourages institutional care.
						The intersection of culture, generation, religion,	Preferences in the care setting	Religious beliefs often serve as a source of comfort and strength for caregivers, helping them cope with the challenges of caregiving. Many view

Supplement I: Data Extraction Table

						and migration		caregiving as part of their fate or a test of faith, though some experience ambivalence, perceiving it at times as divine punishment.
						The intersection of culture, generation, and migration	Preferences in the care setting	Institutional care is stigmatized, creating a significant emotional barrier. Cultural concerns, such as unmet religious needs, continue to limit the acceptance of external support. Turkish-origin view caregiving as a private family responsibility, often leading to isolation and delayed recognition of the need for professional care. While first-generation post-migrants tend to resist external support, second and third generations face growing tensions as they juggle caregiving with their own careers, family roles, and personal lives.
						The intersection of culture and migration	Other daily preferences connected to sexuality	Native-language self-help groups can be effective in raising awareness about dementia and caregiving, particularly for first-generation post-migrants. While native-language services are essential for these families now, their

Supplement I: Data Extraction Table

						The intersection of language and migration	Expectations in nursing homes/care institutions	importance may decrease over time as future generations become more proficient in the local language.
**[22] Wong et al. (2024)** Living with dementia: Exploring the intersections of culture, race, and dementia, stigma, The University of British Columbia, Canada	Semi-structured interviews<br><br>English, peer-reviewed article	Exploring how social location, particularly in relation to culture and race, effects or shapes the experience of stigma and discrimination	N= 10 people living with dementia	South Asian, Spanish-speaking and Chinese in the Vancouver area of BC	Community setting, walk-in clinics, and primary healthcare and support services	The intersection of tradition and migration<br><br>The intersection of tradition and migration	Food preferences<br><br>Other daily preferences about language	Dementia can lead to cultural loss. The study discusses how dementia affects not only memory but also cultural identity and family bonds.<br><br>Stigma in dementia care systems can manifest through language barriers. English-only practices unintentionally exclude non-native speakers, particularly those with cognitive impairments, reinforcing systemic stigma.

Supplement I: Data Extraction Table

REFERENCES

1. Antelius, E. and C. Plejert, *Ethnoculturally-profiled care: Dementia caregiving targeted towards Middle Eastern immigrants living in Sweden.* Anthropology & Aging, 2016. **37**: p. 9-26.

2. Chaouni, S.B., A. Claeys, and L. De Donder, *The influence of religion on the care experiences of family carers of older migrants with dementia in Belgian cities.* Mental Health, Religion & Culture, 2024: p. 1-16.

3. Czapka, E.A. and M. Sagbakken, *"It is always me against the Norwegian system." barriers and facilitators in accessing and using dementia care by minority ethnic groups in Norway: a qualitative study.* BMC Health Serv Res, 2020. **20**(1): p. 954.

4. Dilworth-Anderson, P. and B.E. Gibson, *The cultural influence of values, norms, meanings, and perceptions in understanding dementia in ethnic minorities.* Alzheimer Disease and Associated Disorders, 2002. **16**(SUPPL. 2): p. S56-S63.

5. Hossain, M.Z. and H.T.A. Khan, *Barriers to access and ways to improve dementia services for a minority ethnic group in England.* J Eval Clin Pract, 2020. **26**(6): p. 1629-1637.

6. Im, E.-O., et al., *Attitudes toward Alzheimer's disease and dementia caregiving and health outcomes: Racial and ethnic differences.* Geriatric Nursing, 2022. **48**: p. 296-302.

7. Kong, E.H., J.A. Deatrick, and L.K. Evans, *The experiences of Korean immigrant caregivers of non-English-speaking older relatives with dementia in American nursing homes.* Qual Health Res, 2010. **20**(3): p. 319-29.

8. Lawrence, V., et al., *Threat to Valued Elements of Life: The Experience of Dementia Across Three Ethnic Groups.* The Gerontologist, 2011. **51**: p. 39-50.

9. Lee Casado, B., et al., *The Experience of Family Caregivers of Older Korean Americans With Dementia Symptoms.* Clinical Gerontologist, 2015. **38**(1): p. 32-48.

10. Leszko, M. and D.J. Allen, *Caring From a Distance: Experiences of Polish Immigrants in the United States Providing Care to Parents With Dementia Overseas.* J Gerontol B Psychol Sci Soc Sci, 2024. **79**(3).

11. Lewis, J.P., et al., *"Making Sense of a Disease That Makes No Sense": Understanding Alzheimer's Disease and Related Disorders Among Caregivers and Providers Within Alaska Native Communities.* Gerontologist, 2021. **61**(3): p. 363-373.

12. Liu, D., et al., *Reexamining the relationships among dementia, stigma, and aging in immigrant Chinese and Vietnamese family caregivers.* J Cross Cult Gerontol, 2008. **23**(3): p. 283-99.

13. Martinez, I.L., et al., *Experience of Alzheimer's Disease Family Caregivers in a Latino Community: Expectations and Incongruences in Support Services.* Journals of Gerontology Series B: Psychological Sciences & Social Sciences, 2022. **77**(6): p. 1083-1093.

14. NÆSs, A. and B. Moen, *Dementia and migration: Pakistani immigrants in the Norwegian welfare state.* Ageing and Society, 2014. **35**(8): p. 1713-1738.

15. Nkimbeng, M., et al., *The Immigrant Memory Collaborative: A Community-University Partnership to Assess African Immigrant Families' Experiences with Dementia.* Int J Environ Res Public Health, 2022. **19**(7).

16. Ramsay, S.C., et al., *Migrant caregiving for family members with mild cognitive impairment: an ethnographic study.* Contemp Nurse, 2017. **53**(3): p. 322-334.

17. Richardson, V.E., et al., *At the intersection of culture: Ethnically diverse dementia caregivers' service use.* Dementia (London), 2019. **18**(5): p. 1790-1809.

18. Sagbakken, M., R.S. Spilker, and R. Ingebretsen, *Dementia and Migration: Family Care Patterns Merging With Public Care Services.* Qual Health Res, 2018. **28**(1): p. 16-29.

19. Sagbakken, M., R. Ingebretsen, and R.S. Spilker, *How to adapt caring services to migration-driven diversity? A qualitative study exploring challenges and possible adjustments in the care of people living with dementia.* PLoS One, 2020. **15**(12): p. e0243803.

20. Shanley, C., et al., *A qualitative study into the use of formal services for dementia by carers from culturally and linguistically diverse (CALD) communities.* BMC Health Services Research, 2012. **12**(1): p. 354.

21. Tezcan-Güntekin, H., *Stärkung der Selbstmanagement-Kompetenzen pflegender Angehöriger türkeistämmiger Menschen mit Demenz*, in *Faculty of Public Health*. 2018, Bielefeld University: Bielefeld.

22. Wong, K.L.Y., G. Johnson, and D. O'Connor, *Living with dementia: Exploring the intersections of culture, race, and dementia, stigma.* Dementia, 2024. **23**(6): p. 1001-1020.
